# Relação da Ascensão Matinal da Pressão Arterial com a Hipertrofia Ventricular Esquerda em Hipertensos Obesos

**DOI:** 10.36660/abc.20230050

**Published:** 2023-09-19

**Authors:** Natascha Gonçalves Francisco Palmeira, Henrique Tria Bianco, Maria Teresa Nogueira Bombig, Fernando Focaccia Povoa, Francisco A. H. Fonseca, Maria Cristina Izar, José Marcos Thalenberg, Braulio Luna, Fabiane Marui, Simone Matheus Fischer, Celso Amodeo, Dilma do Socorro Moraes de Souza, Rui Povoa

**Affiliations:** 1 Escola Paulista de Medicina Universidade Federal de São Paulo São Paulo SP Brasil Escola Paulista de Medicina da Universidade Federal de São Paulo (EPM/UNIFESP), São Paulo, SP – Brasil; 2 Universidade Federal de São Paulo São Paulo SP Brasil Universidade Federal de São Paulo, São Paulo, SP – Brasil

**Keywords:** Hipertrofia Ventricular Esquerda, Monitorização Ambulatorial da Pressão Arterial, Obesidade

## Abstract

**Fundamento:**

O aumento do peso frequentemente desencadeia mecanismos que elevam a pressão arterial. A obesidade causa mudanças estruturais no miocárdio, incluindo aumento da massa ventricular, dilatação atrial, bem como disfunções diastólicas e sistólicas. Além disso, variações pressóricas nos hipertensos obesos, como a ascensão matinal (AM), podem ter relevância clínica na prevenção dos eventos cardiovasculares. A AM da pressão arterial é um fenômeno fisiológico, que quando elevada pode ser considerada um fator de risco independente para eventos cardiovasculares.

**Objetivo:**

Avaliar valores da elevação da AM e sua associação com a hipertrofia ventricular esquerda (HVE) e com o Descenso do Sono (DS) em obesos e não obesos hipertensos.

**Métodos:**

Estudo transversal que avaliou medidas pressóricas à monitorização ambulatorial da pressão arterial (MAPA) e a presença de HVE, avaliada pela ecocardiografia, em 203 pacientes hipertensos em tratamento ambulatorial, separados em dois grupos: 109 não obesos e 94 hipertensos obesos. O nível de significância adotado foi de 0,05 em testes bicaudais.

**Resultados:**

A AM acima de 20 mmHg à MAPA foi detectada em 59,2% dos pacientes do grupo “não obesos” e em 40,6% no grupo “obesos”. A HVE foi encontrada em 18,1% no grupo dos não-obesos e em 39,3% no grupo de obesos, p<0,001. No grupo “obesos” foi observado que AM >16 mmHg esteve associada à HVE, com [razão de prevalência: 2,80; IC95% (1,12–6,98), p=0,03]. Para o grupo dos “não obesos”, o ponto de corte da AM para essa associação foi >22 mmHg.

**Conclusão:**

A AM elevada associou-se positivamente com HVE, com comportamento peculiar na população de hipertensos e obesos.

## Figura Central

**Figure:**
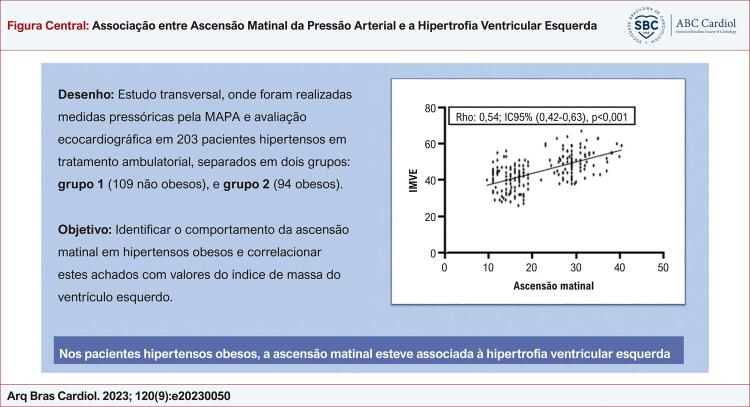
: Associação entre Ascensão Matinal da Pressão Arterial e a Hipertrofia Ventricular Esquerda

MAPA: monitorização ambulatorial da pressão arterial.

## Introdução

Obesidade e sobrepeso na idade adulta estão associados à redução na expectativa de vida e aumento na mortalidade prematura.^1^Vários mecanismos interrelacionados desempenham papel importante no desenvolvimento de hipertensão arterial na obesidade, muitas vezes contribuindo para danos em órgãos-alvo, incluindo doenças cardiovasculares e doenças renais crônicas. Comorbidades associadas à obesidade são facilitadas ou contribuem para uma prevalência alta de hipertensão arterial na população obesa,^2,3^ por mecanismos que incluem a resistência insulínica, inflamação, estresse oxidativo, desbalanço do sistema nervoso autônomo, e atividade do sistema renina-angiotensina aldosterona (SRAA).^4-7^

Além disso, a obesidade pode causar modificações estruturais no coração, incluindo aumento volumétrico atrial e hipertrofia ventricular esquerda (HVE), associando-se às disfunções sistólicas e diastólicas. O aumento ponderal desencadeia mecanismos que elevam a pressão arterial (PA). Desta forma, variações pressóricas nos hipertensos obesos aumentam o risco de eventos cardiovasculares.^8,9^

Variações pressóricas mensuráveis, incluindo o descenso do sono (DS) e a ascensão matinal (AM), podem fornecer valiosas informações prognósticas, sobretudo por suas relações com a atividade do sistema nervoso autônomo e pelo envolvimento no ciclo circadiano.^10^ A AM é considerada uma resposta fisiológica neural e humoral à ativação do sistema simpático, entretanto, a elevação matinal pressórica precoce tem mostrado implicações negativas sobre os desfechos cardiovasculares, estando associado a eventos como o acidente vascular cerebral (AVC), infarto do miocárdio e morte súbita.^11,12^

Há considerações na literatura de que a AM pode ser uma manifestação de uma síndrome composta por alterações hemodinâmicas e aterotrombóticas, enfrentada pelas diferenças pressóricas entre o despertar e o período de sono.^13^

Assim, o objetivo de nosso estudo foi identificar o comportamento da AM em hipertensos obesos, correlacionar estes achados com valores do índice de massa do ventrículo esquerdo (IMVE) e comparar a intensidade da AM e do DS nos pacientes hipertensos obesos com a observada no grupo de hipertensos não obesos.

## Métodos

### Desenho do estudo

Estudo do tipo transversal realizado em centro universitário. Foram realizadas medidas pressóricas de 203 pacientes hipertensos em tratamento ambulatorial, separados em dois grupos: grupo 1 (109 não obesos), e grupo 2 (94 obesos). O estudo foi aprovado pelo comitê de ética em pesquisa da universidade, e todos os participantes ou seus representantes legais, assinaram o termo de consentimento livre e esclarecido.

### Parâmetros avaliados

Foram analisados dados da monitorização ambulatorial da PA (MAPA) em período de 24 horas e parâmetros do ecocardiograma bidimensional com *Doppler* (ECO).

### MAPA

Os aparelhos de MAPA foram usados para o registro da PA sistólica (PAS) e diastólica (PAD) em intervalos de 15-30 minutos na vigília durante o dia, e de 30-60 minutes durante o sono no período noturno. Os aparelhos foram instalados no braço não dominante dos participantes, conforme orientações de diretriz nacional.^14^

Os participantes também foram solicitados a anotar em diário os horários em que foram dormir e acordaram. Foram analisadas as médias da PAS e PAD no período total; na vigília; durante o sono; cargas pressóricas sistólicas e diastólicas; DS; e AM da PAS. A AM foi calculada pela diferença entre a PAS matinal (média das pressões nas primeiras duas horas após o despertar) e a menor PAS durante o sono (média da pressão mais baixa e das pressões imediatamente antes e após a mais baixa).

Todos os parâmetros foram comparados com estratos de normalidade, incluindo a presença ou ausência do DS, que foi segmentado: a) presente (queda da PA durante o sono entre 10 a 20% em relação à vigília); b) atenuado (queda da PA 0-10% durante o sono); c) invertido (PA no sono mais elevada que na vigília). Foi utilizado o monitor *Spacelabs*^®^ 90207, validado pela *Association for the Advancement of Medical Instrumentation* (organização fundada em 1965, destinada a promover o desenvolvimento, uso seguro e eficaz de tecnologia médica) e instalado no mesmo braço onde foi aferida a pressão de consultório.^15^

### Ecocardiografia bidimensional com mapeamento de fluxo colorido

Este exame foi realizado por investigador cardiologista devidamente habilitado, e que desconhecia as características basais dos participantes. Os estudos ultrassonográficos obedeceram aos preceitos da *American Society of Echocardiography* e da *European Association of Echocardiography.* Foram obtidos os seguintes parâmetros: diâmetro sistólico do ventrículo esquerdo (DSVE), diâmetro diastólico final do ventrículo esquerdo (DDVE), espessura do septo interventricular (SIV), e espessura da parede posterior do ventrículo esquerdo (PPVE). Também foram obtidos os volumes diastólicos e sistólicos finais, porcentual de encurtamento diastólico, e fração de ejeção, aferida pelo método do cubo. Para o cálculo da massa ventricular esquerda, a presença de HVE foi detectada pelo IMVE de acordo com a fórmula de Devereux e ajustada pela superfície corporal: $\text{ massa do VE }=0,8\times \{1,04[(SIV+DDVE+\text{ PPVED }{)}^{3}-(\text{ DDVE }{)}^{3}\}+0,6g$ .^16^A massa do VE foi associada à superfície corpórea e indexada (elevada à 2,7), para ajustes das dimensões do coração às variações antropométricas.^17^O equipamento utilizado foi o *Image Point Hx*- HP^®^, com o transdutor linear número 04 Hertz.

### Análise estatística

O cálculo do tamanho amostral foi realizado *a priori* para um poder de 0,95 (1- β); erro α em 5%, estimando um tamanho de efeito de 0,3. Desta forma, encontramos o valor total da amostra em 134 participantes. O teste de qualidade do ajuste foi utilizado para determinar se os dados da amostra eram consistentes com uma distribuição hipotética. Os pacientes eram provenientes de um ambulatório de especialidade, em centro universitário. Para análise do pressuposto de normalidade utilizamos o teste de Kolmogorov–Smirnov. As variáveis contínuas com distribuição normal foram representadas pelas médias e desvio-padrão e as que não apresentavam distribuição normal foram expressas pelas medianas e intervalos interquartis (IIQ). As variáveis categóricas foram expressas em números absolutos e porcentagens. As variáveis contínuas com distribuição Gaussiana foram comparadas pelo teste t-Student para amostras independentes, e dados não paramétricos pelo teste Mann-Whitney. O teste qui-quadrado (χ^2^) foi utilizado para estudar a associação entre as variáveis qualitativas. A correção de Yates de continuidade foi utilizada para adequar os valores de p do qui-quadrado.

As correlações entre as variáveis de interesse não paramétricas foram avaliadas pelo método de Spearman para a obtenção do índice de correlação (Rho). A variável AM foi dicotomizada para os dois grupos, com análise concomitante dos resíduos. O pressuposto de homocedasticidade foi analisado graficamente (*scatterplot),* e a análise de multicolinearidade foi realizada assumindo fatores de inflação de variância (VIF) inferiores a cinco e o índice de tolerância (IT) inferior a 0,20. O tamanho de efeito (*d-Cohen*) foi calculado pela diferença entre as médias da AM entre os grupos, considerando os desvios-padrões.

Análises de regressão múltipla para a presença de HVE (como variável dependente) foram realizadas usando as seguintes covariáveis: idade, sexo, PAS, PAD, AM e tamanho de átrio esquerdo, para determinar o grau de independência da associação. Por se tratar de estudo transversal, obtivemos as razões de prevalência (RP) pelo teste de Wald. O valor de p bilateral <0,05 foi considerado estatisticamente significativo. Todas as análises foram realizadas utilizando-se o *software* SPSS, versão 26.0 (SPSS Inc.Chicago, IL, EUA) ^®^

## Resultados

**Figura 1:**
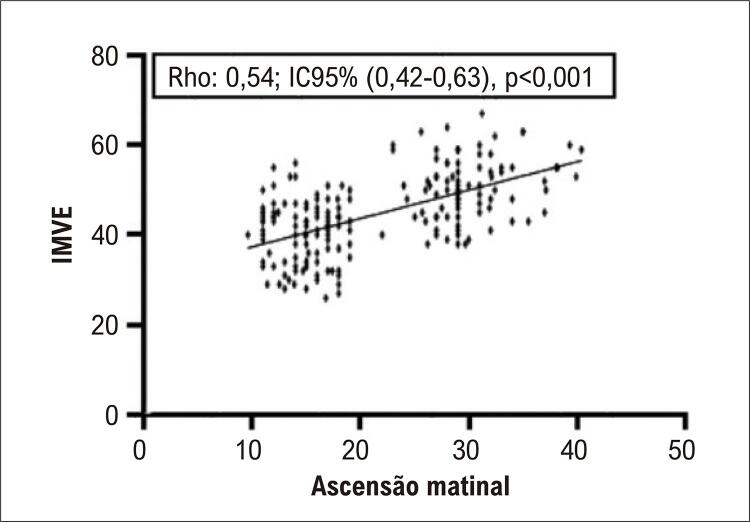
– Correlação entre a ascensão matinal e o índice de massa ventricular esquerda (IMVE). Análise de correlação de Spearman, em dados não paramétricos, com índice (Rho) no intervalo de confiança 95%.

**Figura 2:**
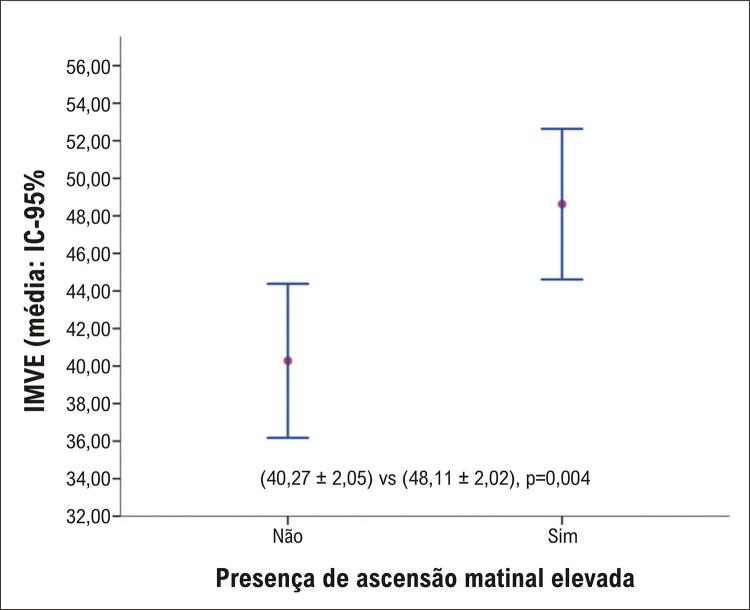
– Box plot das médias com seus respectivos intervalos de confiança (IC) 95% para o índice de massa ventricular esquerda (IMVE) nos grupos com e sem ascensão matinal (AM).

As principais características dos participantes estão descritas na Tabela 1. A prevalência de HVE foi de 38,4% no total de participantes, e 74,3% no grupo dos pacientes com obesidade. Houve diferença significativa entre os grupos obesos *vs* não obesos quanto ao tamanho atrial esquerdo, SIV, PPVE e o IMVE. Pela MAPA, foi observada diferença na AM entre os grupos, sendo a prevalência da AM acima de 20 mmHg, associada à HVE, significativamente maior no grupo de obesos (Tabela 2). O tamanho de efeito, calculado a partir da diferença entre as médias da AM entre grupos foi de 0,40, com poder amostral real de 0,95. Observamos correlação positiva entre o IMVE e a AM, com Rho: [0,54; IC-95% (0,42-0,63), p<0,001], vista também em (Figura 1 e Figura Central). As médias do IMVE com seus respectivos IC95% dos grupos com e sem AM elevada estão apresentadas na Figura 2. Nos hipertensos obesos, a média do IMVE foi de 52,87 ± 13,37 *versus* 40,58 ± 12,29 para os hipertensos não obesos, p<0,001.

**Tabela 1:** – Características da população estudada

	População geral	Grupo não obesos	Grupo obesos	p-valor
n (%)	203	109 (53,7)	94 (46,3)	
**Epidemiológicos**				
Idade, md (IIQ)	59 (50-67)	62 (18-87)	58 (18-79)	0,28
Sexo masculino, n (%)	57 (28,1)	35 (32,1)	22 (23,4)	0,17
Sexo feminino, n (%)	146 (71,9)	74 (67,8)	72 (76,5)	0,19
IMC, md (IIQ)	27,48 (17,31-50,43)	23,83 (17,31-29,48)	38,33 (30,12-50,43)	<0,001
Diabetes, n (%)	63 (31,1%)	19 (17,4%)	44 (46,8%)	<0,001
**Ecocardiografia**				
Átrio esquerdo, (cm); m±dp	3,66 ± 0,55	3,52 ± 0,61	3,82 ± 0,44	<0,001
Septo Interventricular, (cm); m±dp	0,97± 0,17	0,92 ± 0,16	1,02 ± 0,16	<0,001
Parede Posterior, (cm); m±dp	0,92 ± 0,13	0,88 ± 0,14	0,96 ± 0,10	<0,001
DDVE, (cm); m±dp	4,95 ± 0,53	4,81 ± 0,49	5,11 ± 0,53	<0,001
IMVE^2,7^, m±dp	46,27 ± 14,17	40,58 ± 12,29	52,87 ± 13,37	<0,001
**MAPA**				
PAS 24 h, mmHg; m±dp	126,51 ± 16,11	126,33 ± 18,03	126,71 ± 13,78	0,87
PAD 24 h, mmHg m±dp	74,66 ± 10,71	76,09 ± 11,35	73,08 ± 9,76	0,04
PAS matinal, mmHg m±dp	129,31 ± 19,97	131,01 ± 20,37	127,33 ± 19,42	0,19
PAS vigília, mmHg m±dp	126,37 ± 17,51	126,26 ± 19,46	126,48 ± 15,16	0,92
PAD vigília, mmHg m±dp	74,35 ± 12,44	75,76 ± 13,04	72,80 ± 11,62	0,09
PAS sono, mmHg m±dp	121,22 ± 19,84	120,71 ± 21,64	121,78 ± 17,73	0,38
PAD sono, mmHg m±dp	70,70 ± 12,73	72,19 ± 12,52	69,05 ± 12,83	0,08
Ascenção matinal, mmHg; m±dp	19,38 ± 13,36	22,16 ± 13,30	16,16 ± 12,67	<0,001

Dados contínuos são expressos como média e desvio padrão (m ± dp) ou mediana e intervalo interquartil (md, IIQ), conforme apropriado. As variáveis categóricas são expressas em números absolutos e porcentagens. Comparações entre as médias realizadas pelo teste t Student em amostras independentes e para as variáveis categóricas pelo teste de proporções do qui quadrado de Pearson. IMC: índice de massa corpórea – Peso/Altura;^2^ DDVE: diâmetro diastólico do ventrículo esquerdo; IMVE:^2,7^índice de massa do ventrículo esquerdo; PAS: pressão arterial sistólica; PAD: pressão arterial diastólica. O valor de p foi expresso nas comparações entre os grupos. A massa do ventrículo esquerdo foi calculada pela fórmula proposta por Devereaux e indexada à superfície corpórea e elevada ao exponencial.^2,7^

**Tabela 2:** – Presença da ascensão matinal (AM) associada à hipertrofia ventricular esquerda (HVE) entre os grupos estudados

	Ascenção Matinal	Ausência de HVE	Presença de HVE	p-valor

		n (%)	n (%)	
Não obesos	AM ausente	44 (80,0)	11 (20,0)	0,80
	AM presente	45 (83,3)	9 (16,7)	
Obesos	AM ausente	27 (47,4)	30 (52,6)	0,031
	AM presente	9 (24,3)	28 (75,7)	

Os dados são expressos em valor absoluto e frequência, n (%). As comparações entre grupos foram realizadas pelo teste do qui-quadrado de Pearson, com seus respectivos valores de significância (p-valor). AM: ascensão matinal; HVE: hipertrofia ventricular esquerda. AM presente: acima de 20 mmHg.

Em análise de regressão linear, um valor de AM >16 mmHg foi o que melhor se associou com a presença de HVE para o grupo dos obesos, em contraste com o valor >22 mmHg para o grupo dos pacientes não obesos. Na regressão logística binária, onde os valores da AM foram dicotomizados, a RP entre a AM e a HVE foi [RP: 2,80; IC95% (1,12–6,98)], p=0,03 e com ponto de corte da AM >16 mmHg para o grupo dos pacientes hipertensos obesos, considerando os necessários ajustes para os possíveis elementos de confusão.

Quanto ao comportamento do DS, no grupo dos pacientes obesos, 73% daqueles que tiveram AM elevada, apresentavam DS fisiológico (queda da PA maior que 10% para este período). Em contrapartida, no grupo dos pacientes não obesos, o DS associado ao aumento da AM esteve presente em 66,7%, (Tabela 3). Destacamos ainda, e de forma esperada, observamos um maior número de agentes anti-hipertensivos utilizados para os pacientes pertencentes ao grupo dos obesos (Tabela 4).

**Tabela 3:** – Associação entre a presença da ascensão matinal (AM) com o padrão de descenso do sono nos grupos estudados

	Padrão de Descenso do sono	Ascenção Matinal ausente	Ascenção Matinal presente	p-valor

		n (%)	n (%)	
Não obesos	Descenso presente	24 (43,6)	36 (66,7)	0,03
	Descenso atenuado	22 (40,0)	15 (27,8)	
	Descenso invertido	9 (16,4)	3 (5,5)	
Obesos	Descenso presente	25 (43,9)	27 (73,0)	0,02
	Descenso atenuado	24 (42,1)	8 (21,6)	
	Descenso invertido	4 (7,0)	2 (5,4)	

O Descenso do sono foi estratificado em: a) presente (queda da PA durante o sono entre 10 a 20% em relação à vigília); b) atenuado (queda da PA >0% e inferior a 10% durante o sono); c) invertido (PA no sono mais elevada que na vigília).

**Tabela 4:** – Número de medicações anti-hipertensivas entre os grupos estudados

Grupo e Medicamentos							

		Número de Medicamentos					Total

		0	1	2	3	4	
Grupo	Não obesos	31	21	36	13	8	109
	Grupo obesos	12	7	30	31	14	94
**Total**		43	28	66	44	22	203

Distribuição entre os grupos (não obesos e obesos), quanto ao número de fármacos anti-hipertensivos utilizados.

## Discussão

Nosso estudo foi delineado para a identificação de prevalência da AM em pacientes obesos hipertensos, que apresentavam evidências ecocardiográficas de HVE, consistente com recomendações de diretrizes de manejo da hipertensão e prevenção de complicações.^18,19^ A AM é uma métrica pressórica de simples aferição, composta pelas diferenças de médias da PA entre o despertar e o período de sono.

A prevalência de obesidade no mundo industrializado vem apresentando aumentos constantes, atingindo níveis alarmantes nas últimas décadas. Estima-se que parcela significativa dos casos de hipertensão guarde alguma relação com a obesidade, e que obesos tenham maior probabilidade de desenvolver hipertensão. A obesidade, a hipertensão arterial e a HVE são importantes fatores de risco cardiovascular. Desta forma, novos biomarcadores têm sido investigados, objetivando melhorar os aspectos preditivos para os desfechos cardiovasculares maiores, ou mesmo sobre os desfechos substitutos.

Níveis pressóricos normalmente seguem um padrão em que a PA é mais baixa durante o sono quando comparada com os valores da vigília. Um aumento na PA durante a transição do sono para o despertar é chamado de AM. Os mecanismos vasculares que levam à AM excessiva e suas implicações fisiopatológicas não estão totalmente elucidados, com a primeira evidência sobre a relação dessa variável pressórica com eventos cardiovasculares relatada em 2003, por Kario et al.,^20^ que observaram associação independente com infartos cerebrais silentes. No estudo Ohasama, conduzido por Metoki et al.,^21^ foi apontada associação positiva e significativa da AM com o AVC hemorrágico.^21^ A relação entre AM e desfechos cardiovasculares esteve presente em outro ensaio, que investigou essa associação em pacientes com DS.^22^Li et al.,^23^ ao avaliarem um extenso banco de dados (*International Database on Ambulatory Blood Pressure*), com 5645 participantes provenientes de oito países, evidenciaram que a AM é preditiva de eventos cardiovasculares, particularmente AVC em asiáticos e eventos coronários em europeus.^23^ O estudo de Pierdomenico et al.^24^ demonstrou que AM acentuada é preditora de AVC em idosos em uso de anti-hipertensivos com DS presente.^24^ Entretanto, no estudo de Verdecchia et al.^25^ com 3012 pacientes inicialmente não tratados, os alocados no quartil superior da AM tiveram o menor risco para todos os eventos cardiovasculares de relevância.^25^ A discrepância nesses resultados deve-se, provavelmente, a inúmeros fatores como heterogeneidade das populações, presença de fatores confundidores e pela falta de um ponto de corte específico para definir AM elevada. Idade média, prevalência de hipertensão, terapia anti-hipertensiva, duração do seguimento, avaliação do impacto do DS, e etnia tenderam a diferir entre os estudos. Uma revisão sistemática e metanálise reuniu os dados da AM de 14133 indivíduos de sete estudos longitudinais, com duração média de acompanhamento em 7,1 anos. Ficou demonstrado que a AM excessivamente elevada estaria associada a maior risco de mortalidade geral. Em pacientes com AM elevada, observou-se uma tendência de aumento do risco para a mortalidade geral, AVC e eventos cardiovasculares totais, porém sem significância estatística.^26^ Metanálise conduzida por Sheppard et al.^27^ demonstrou que, ao considerarmos a AM como uma variável contínua, a qual tem maior poder para detectar associação, um aumento de 10 mmHg estava associado ao maior risco de AVC.^27^Resultados semelhantes foram apresentados por Kario et al.,^20^ citado previamente, ao avaliarem a AM como variável contínua, com demonstração de que aumentos desse biomarcador esteve associado ao maior risco de AVC.

Um ponto de corte universal para definir AM anormal ainda não foi estabelecido. Hoshide et al.,^28^ no estudo ARTEMIS demonstraram que a AM era maior em japoneses hipertensos que em indivíduos europeus hipertensos, mesmo após ajustes para idade e níveis médios da PA em 24 horas. Essa diferença permaneceu significativa após considerar as diferenças no DS.^28^Marcadores de doença cardíaca hipertensiva, que incluem aumento do IMVE, HVE e uma relação A/E mais baixa (medida de disfunção diastólica) foram associados a acréscimos exacerbados da PA matinal.^29-32^

As dificuldades na redução do peso corpóreo e no controle de medicações para garantir a adesão à terapêutica e mitigar as doenças crônicas degenerativas são os principais desafios da equipe multiprofissional. Encontrar instrumentos ou indicadores de associação pode ser de utilidade para a predição dos eventos, notadamente na população hipertensa obesa. A obesidade, antes um mero fator de risco entre tantos, deve ser considerada uma doença crônica e um problema de saúde pública, demandando investimentos em pesquisas e tratamentos nos próximos anos. Considerado agora um fator de risco independente para as doenças cardiovasculares, o excesso de adiposidade corporal é fator predisponente no desenvolvimento de hipertensão nestes pacientes. Pelo número crescente de hipertensos no mundo, estudos epidemiológicos voltados à compreensão do comportamento pressórico são cada vez mais discutidos, tendo em vista correlações significativas entre a variabilidade pressórica (indicadores como os tipos de AM, DS, entre outros) e o desenvolvimento de lesões de órgãos-alvo.

A maioria dos mecanismos fisiológicos segue um padrão circadiano, determinado por uma complexa interação do nosso “relógio biológico” com fatores ambientais e comportamentais. Muitos desses mecanismos têm efeito direto sobre o sistema cardiovascular e contribuem para o aumento da PA. Em particular, alterações na atividade do sistema nervoso autônomo, notadamente as relacionadas ao aumento da atividade simpática, parecem ser o principal fator subjacente à AM.^33^Wanthong et al.^34^ descreveram que valores da PAS ao despertar, apresentavam correlações com o IMVE, assim como para o risco residual em eventos cardiovasculares. Além disso, apontaram a importância da medida de PA no período do sono como marcador independente de lesões em órgãos-alvo.^34^

Em nossa população estudada, observamos que no grupo de hipertensos obesos, valores de AM acima de 16 mmHg apresentavam correlação linear positiva com a HVE, ponto de corte que definiu um melhor desempenho entre a sensibilidade e especificidade. Provavelmente, e de forma especulativa, a obesidade potencializa os efeitos agressivos da AM ou é um fator adjuvante do risco. A coexistência de obesidade e hipertensão agrega probabilidade de complicações cardiovasculares, tendo em vista que o aumento do peso amplifica o risco de outras doenças como diabetes e doença renal cronica.^35^De fato, observamos que 46,8% da população obesa era também diabética, *versus* 17,4% no grupo de não obesos, p<0,001. Aproximadamente 33% dos pacientes não obesos (grupo 1) com HVE e diabetes tiveram a AM presente, enquanto no grupo dos obesos (grupo 2), encontramos a taxa de 80%.

Há evidências de que as medidas pressóricas aferidas pela MAPA são as melhores preditoras para os desfechos, incluindo mortalidade geral, tendo aspecto acurado quando comparadas às medidas de consultório.^36^Em nossos dados, a média da AM foi de 16,1±12,6 mmHg nos hipertensos obesos, em contraste com os hipertensos não obesos, cuja média foi de 22,1±13,3 mmHg, p<0,001. Destacamos a associação positiva entre a AM>16 mmHg com a presença de HVE no grupo dos obesos, especulando, portanto, que um menor valor desse biomarcador possa ser utilizado e estratificado para esta população.

A despeito de poucas evidências referenciadas sobre a AM na população obesa, Amodeo et al.^37^ indicaram para a necessidade de mais estudos populacionais para avaliar o impacto da AM, e a definição de um valor de corte para a AM.^37^ Em nossa coorte, a melhor associação com a HVE foi com a AM acima de 16 mmHg para o grupo de indivíduos obesos. Desta forma, notamos que a obesidade pode ter influência expressiva nos valores pressóricos e nas alterações estruturais do ventrículo esquerdo. Nos hipertensos obesos, a média do IMVE foi maior quando comparado aos hipertensos não obesos. Em estudo prospectivo^38^ com 433 participantes, a obesidade e a hipertensão arterial foram os principais fatores determinantes do remodelamento ventricular esquerdo e do surgimento da HVE. Por meio de interação significativa entre obesidade e hipertensão arterial, os obesos frequentemente desenvolvem hipertensão e sobrecarga pressórica, exercendo efeito exponencial na prevalência da HVE. Estudos epidemiológicos de grande porte mostraram que a hipertensão arterial era fator crucial para o remodelamento concêntrico do ventrículo esquerdo e da HVE concêntrica, e de forma independente da obesidade.^38-40^ Além disso, há evidências que o fator inibidor da migração de macrófagos, citocina envolvida em amplo espectro de eventos no sistema imune, estaria associado à disfunção endotelial e ao remodelamento ventricular esquerdo.^41^

Desta forma, destacamos que a AM na população hipertensa e obesa pode ser um fator associado e ter comportamento peculiar, e mensurações pressóricas matinais seriam mais sensíveis para detectar o impacto da variabilidade da PA no risco cardiovascular. Em análise de nossas observações, sugerimos a composição de uma estratégia otimizada, incluindo o registro de todas as variáveis pressóricas, e especulamos que a AM, sobretudo em populações específicas, como a dos hipertensos obesos, possa estar associada a lesões estruturais do miocárdio.

## Conclusão

Nos pacientes hipertensos obesos, a AM elevada esteve positivamente associada à HVE, com limiar de correlação abaixo dos observados no grupo de pacientes não obesos. Essa análise de PA pela MAPA evidencia que valores pressóricos aferidos no início da manhã estiveram associados a danos aos órgãos-alvo, notadamente expressando a HVE. Portanto, os resultados de nossa análise podem ser úteis para avaliar o risco residual em subgrupos de pacientes, a despeito e além do risco avaliado pela medição pressórica convencional.

### Limitações do estudo

Assim como em estudos populacionais, a dificuldade para o achado de AM em obesos provém das mensurações ideais, principalmente no período do sono, pela adequação do manguito e complicações relacionadas a possíveis distúrbios nesse período. Por se tratar de estudo observacional, não podemos inferir causalidade entre as variáveis estudadas. Ademais, nossos dados são provenientes de centro único e possíveis vieses de seleção precisam ser considerados. Os horários de sono e vigília foram autorrelatados. Existe a possibilidade de que esses horários tenham sido relatados erroneamente por alguns participantes, resultando em possíveis erros nas estimativas do aumento matinal da PA. Outra possível limitação deve-se ao fato de não analisarmos outras variáveis, como a qualidade do sono, e talvez um número amostral maior, além da validação externa de nossos achados.
