# Supplementary material for: Relação da Ascensão Matinal da Pressão Arterial com a Hipertrofia Ventricular Esquerda em Hipertensos Obesos
Source: Arq Bras Cardiol. 2023 Sep 19;120(9):e20230050. [Article in Portuguese] doi: 10.36660/abc.20230050 (PMC10519347; doi:10.36660/abc.20230050)
Supplement: Translation

# Translation: Association between Morning Surge and Left Ventricular Hypertrophy in Obese Hypertensive Patients

**Authors:** Natascha Gonçalves Francisco Palmeira, Henrique Tria Bianco, Maria Teresa Nogueira Bombig, Fernando Focaccia Povoa, Francisco A. H. Fonseca, Maria Cristina Izar, José Marcos Thalenberg, Braulio Luna, Fabiane Marui, Simone Matheus Fischer, Celso Amodeo, Dilma do Socorro Moraes de Souza, Rui Povoa

**Affiliations:** 1 Brasil Escola Paulista de Medicina da Universidade Federal de São Paulo (EPM/UNIFESP), São Paulo, SP – Brasil; 2 Brasil Universidade Federal de São Paulo, São Paulo, SP – Brasil

**Keywords:** Hypertrophy, Left Ventricular, Blood Pressure Monitoring, Ambulatory, Obesity

## Abstract

**Background:**

Weight gain can trigger mechanisms that increase blood pressure. Nevertheless, obesity causes structural changes in the myocardium, including increased ventricular mass, atrial dilatation, and diastolic and systolic dysfunction. Additionally, blood pressure variations, like morning surge (MS) in obese hypertensive patients may have clinical relevance in cardiovascular events. Although morning blood pressure surge is a physiological phenomenon, excess MS can be considered an independent risk factor for cardiovascular events.

**Objective:**

To evaluate MS values and their association with left ventricular hypertrophy (LVH) and nocturnal dipping (ND) in obese and non-obese hypertensive patients.

**Methods:**

A cross-sectional study that evaluated BP measurements by ambulatory blood pressure monitoring (ABPM) and the presence of LVH by echocardiography in 203 hypertensive outpatients, divided into two groups: 109 non-obese and 94 obese hypertensives patients. The significance level was set at 0.05 in two-tailed tests.

**Results:**

A MS above 20 mmHg by ABPM was detected in 59.2% of patients in the non-obese group and 40.6% in the obese group. LVH was found in 18.1% and 39.3% of patients in the non-obese and obese groups, respectively, p<0.001. In the “obese group”, it was observed that a MS>16 mmHg was associated with LVH, [prevalence ratio: 2.80; 95%CI (1.12–6.98), p=0.03]. For the non-obese group, the cut-off point of MS for this association was >22 mmHg.

**Conclusion:**

High MS was positively associated with LVH, with a particular behavior in the hypertensive obese group.

## Introduction

Adult overweight and obesity are associated with reduced life expectancy and increased premature death.^1^ Several interrelated mechanisms play an important role in the development of arterial hypertension in obesity, often contributing to target organ damage, including cardiovascular diseases and chronic kidney disease. Obesity-related comorbidities are facilitated by and contribute to a high prevalence of arterial hypertension in the obese population,^2,3^ by mechanisms including insulin resistance, inflammation, oxidative stress, autonomous nervous system, and activity of the renin-angiotensin-aldosterone system (RAAS).^4-7^

In addition, obesity may cause structural changes in the heart, including increased left atrial volume and left ventricular hypertrophy (LVH), which are associated with systolic and diastolic dysfunction. The increase in body weight triggers mechanisms that increase blood pressure (BP), and BP variations in obese hypertensive subjects increase the risk of cardiovascular events.^8,9^

Measurable BP variations, such as nocturnal dipping (ND) and morning surge (MS), can provide valuable prognostic information, especially due to relationship with the autonomous nervous system activity and the circadian cycle.^10^ The MS is considered a neural and humoral physiological response to activation of the sympathetic system; however, early BP elevation in the morning seem to have negative implications for cardiovascular outcomes, with association with events like cerebrovascular accident, myocardial infarction and sudden cardiac death.^11,12^

The literature has proposed that MS in BP may be a manifestation of a hemodynamic and atherothrombotic syndrome, marked by differences in BP between post-awakening and night-trough.^13^

Therefore, the aim of this study was to characterize morning BP surge in obese hypertensive individuals, to correlate it with left ventricular mass index (LVMI) and to compare the intensity of MS and ND in obese hypertensive patients with that observed in non-obese hypertensive patients.

## Methods

### Study design

This was a cross-sectional study conducted in a university center. BP measurements of 203 hypertensive outpatients were taken, and patients were divided into two groups, group 1 (109 non-obese patients) and group 2 (94 obese patients). The study protocol was approved by the ethics committee of the university and all participants or legal representatives signed the informed consent form.

### Outcome measures

Parameters of 24-hour ambulatory BP monitoring (ABPM) and two-dimensional echocardiography with Doppler were analyzed.

### ABPM

The ABPM devices were programmed to take systolic BP (SBP) and diastolic BP (DBP) readings every 15-30 minutes at awakening during the day, and every 30-60 minutes during sleep at night. The ABPM devices were worn on the non-dominant arm following the Brazilian national guidelines.^14^

Participants were asked to record the time they went to sleep at night and awoke in the morning. Mean SBP and DBP values from 24-hour monitoring and during sleep, systolic and diastolic BP load, ND, and MS of SBP were analyzed. MS was calculated as the difference between SBP in the morning (mean BP measurements in the first two hours after awakening) and the lowest SBP during sleep (mean of three BP measurements – the lowest reading, the one immediately before it and the one immediately after it).

All parameters were compared with normal ranges, including the presence or absence of MS, and categorized as follows: a) present (10-20% drop in BP from awake to sleep periods), b) attenuated (<10% drop in nighttime BP), c) reverse (nighttime BP greater than daytime BP). The *Spacelabs*^®^ 90207 monitor, validated by the Association for the Advancement of Medical Instrumentation, which is an organization for promoting the development, and safe and effective use of medical technology founded in 1965) was worn on the same arm the office BP measurements were taken.^15^

### Two-dimensional echocardiography with color flow mapping

Two-dimensional echocardiography with color flow mapping was conducted by a trained cardiologist who was unaware of participants’ baseline characteristics. The ultrasound studies followed the American Society of Echocardiography and the European Association of Echocardiography guidelines. The following parameters were analyzed: left ventricular systolic diameter (LVSD), left ventricular end-diastolic diameter (LVEDD), interventricular septal thickness (IVST), and left ventricular posterior wall thickness (LVPWT). In addition, end-diastolic and end-systolic volume, diastolic fractional shortening, and ejection fraction by the cube method. For calculation of the left ventricular mass, LVH was defined by the LVMI using the Deveureux’s formula, and adjusted by body surface: $\text{left ventricular mass }=0.80\{1.04(\text{ IVST }+\text{ LVEDD }+\text{ LVPWT }{)}^{3}-(\text{ LVEDD }{)}^{3}\}+0.6g$ .^16^Left ventricular mass was associated with body surface and indexed (raised to 2.7) for adjustments for cardiac chamber dimensions and anthropometric variation.^17^ The Image Point Hx- HP® machine was used, with a 4Hz linear transducer.

### Statistical analysis

Sample size was first calculated with a power of 0.95 (1-β); α error of 5%, yielding a size effect of 0.3, and hence a sample size of 134 participants. The adjustment quality was assessed to determine whether the sample data were consistent with a hypothetical distribution. Patients were recruited at a specialty outpatient clinic of a university center. The Kolmogorov-Smirnov test was used to verify the normal distribution of the data. Continuous variables with normal distribution were expressed as mean and standard deviation and those without a normal distribution as median and interquartile range (IRQ). Categorical variables were expressed as absolute numbers and percentages. Continuous variables with normal distribution were compared using the t-Student test for independent samples, and non-parametric data with the Mann-Whitney test. The chi-square test (χ^2^) was used to assess the association between qualitative variables. The Yates’ correction for continuity was used to adjust the chi-square p-values.

Correlations between the non-parametric variables were assessed using the Spearman’s rank correlation coefficient rho (Rho) rank correlation. The MS variable was dichotomized into two groups, with concomitant analysis of residues. The homoscedasticity assumption was analyzed graphically (scatterplot) and the multicollinearity analysis was performed variance inflation factors (VIFs) lower than five and a tolerance index (TI) lower than 0.20. The effect size (d-Cohen) was calculated by the difference between mean MS values between the groups, considering the standard deviations.

Multiple regression analysis for the presence of LVH (dependent variable) were performed using the covariables age, sex, SBP, DBP, MS and left atrial size, to determine the degree of independence in the association. As this was a cross-sectional study, the prevalence ratio (PR) was calculated using the Wald test. A two-tailed p<0.05 was considered statistically significant. All analyses were performed using the SPSS software version 26.0 (SPSS Inc. Chicago, IL, EUA) ^®^

## Results

The main characteristics of participants are described in Table 1. The prevalence of LVH was 38.4% in total sample, and 74.3% in the group of obese patients. There was a significant difference between obese and non-obese patients for the left atrial size, IVS, LVPW and LVMI. According to ABPM, there was a significant difference in MS between the groups, with a prevalence of MS above 20mmHg and LVH significantly higher in the obese group (Table 2), associated. The size effect, calculated as the difference between mean MS values between the groups was 0.40, with a power of 0.95. There was a positive correlation between LVMI and MS, with a Rho of [0.54; 95%CI (0.42-0.63), p<0.001] (Figure 1 and Central Illustration). Mean LVMI and respective 95%CI in the groups of subjects with and without an increased MS are shown in Figure 2. In obese hypertensive individuals, mean LVMI was 52.87 ± 13.37 *versus* 40.58 ± 12.29 in non-obese hypertensive individuals (p<0.001).

**Table 1:** – Characteristics of the study population

	General population	Non-obese group	Obese group	p-value
n (%)	203	109 (53.7)	94 (46.3)	
**Epidemiological**				
Age, median (IQR)	59 (50-67)	62 (18-87)	58 (18-79)	0.28
Male sex, n (%)	57 (28.1)	35 (32.1)	22 (23.4)	0.17
Female sex, n (%)	146 (71.9)	74 (67.8)	72 (76.5)	0.19
BMI, median (IQR)	27.48 (17.31-50.43)	23.83 (17.31-29.48)	38.33 (30.12-50.43)	<0.001
Diabetes, n (%)	63 (31.1%)	19 (17.4%)	44 (46.8%)	<0.001
**Echocardiography**				
Left atrium, (cm); mean±SD	3.66 ± 0.55	3.52 ± 0.61	3.82 ± 0.44	<0.001
Interventricular septum, (cm); mean±SD	0.97± 0.17	0.92 ± 0.16	1.02 ± 0.16	<0.001
Posterior wall, (cm); mean±SD	0.92 ± 0.13	0.88 ± 0.14	0.96 ± 0.10	<0.001
DDVE, (cm); mean±SD	4.95 ± 0.53	4.81 ± 0.49	5.11 ± 0.53	<0.001
IMVE^2,7^, mean±SD	46.27 ± 14.17	40.58 ± 12.29	52.87 ± 13.37	<0.001
**MAPA**				
SBP 24 h, mmHg; mean±SD	126.51 ± 16.11	126.33 ± 18.03	126.71 ± 13.78	0.87
DBP 24 h, mmHg mean±SD	74.66 ± 10.71	76.09 ± 11.35	73.08 ± 9.76	0.04
Morning SBP, mmHg mean±SD	129.31 ± 19.97	131.01 ± 20.37	127.33 ± 19.42	0.19
Waking SBP, mmHg mean±SD	126.37 ± 17.51	126.26 ± 19.46	126.48 ± 15.16	0.92
Waking DBP, mmHg mean±SD	74.35 ± 12.44	75.76 ± 13.04	72.80 ± 11.62	0.09
Sleep SBP, mmHg mean±SD	121.22 ± 19.84	120.71 ± 21.64	121.78 ± 17.73	0.38
Sleep DBP, mmHg mean±SD	70.70 ± 12.73	72.19 ± 12.52	69.05 ± 12.83	0.08
Morning surge, mmHg; mean±SD	19.38 ± 13.36	22.16 ± 13.30	16.16 ± 12.67	<0.001

Continuous data are expressed as mean and standard deviation (SD) or median and interquartile range (IQR), as appropriate. Categorical variables were expressed as absolute numbers and percentage. Comparisons between means were performed by the Student’s t-test for independent samples and Pearson’s chi-square test for categorical variables; BMI: body mass index (weight/height^2^); LVDD: left ventricular diastolic diameter; LVMI:^2,7^ left ventricular mass index; SBP: systolic blood pressure; DBP: diastolic blood pressure; P-value was expressed for comparison between the groups. Left ventricular mass was calculated using the Deveureux’s formula, adjusted by body surface and fitted to the exponential.^2,7^

**Table 2:** – Presence of morning rise in blood pressure (morning surge; MS) associated with left ventricular hypertrophy between the studied groups

	Morning surge	Absence of LVH	Presence of LVH	p-value

		n (%)	n (%)	
Non-obese	Absent MS	44 (80.0)	11 (20.0)	0.80
	Present MS	45 (83.3)	9 (16.7)	
Obese	Absent MS	27 (47.4)	30 (52.6)	0.031
	Present MS	9 (24.3)	28 (75.7)	

Data expressed as absolute values and frequency, n (%); between-group comparisons were performed by Pearson’s chi-square test and respective significance values (p-value); MS: morning surge; LVH: left ventricular hypertrophy; Morning surge > 20 mmHg.

**Figure 1:** – Correlation between morning surge and left ventricular mass index; 95% confidence interval for Spearman’s (Rho)rank correlation coefficient.

**Figure 2:** – Boxplot of mean left ventricular mass index (LVMI) and respective 95% confidence intervals in the groups with and without morning blood pressure surge.

In linear regression analysis, a MS value > 16mmHg showed the strongest association with the presence of LVH in the obese group, in contrast with a MS > 22 mmHg in the non-obese group. In binary logistic regression, in which the MS values were dichotomized, the relationship between the PR between MS and LVH was [PR: 2.80; 95%CI (1.12–6.98)], p=0.03 with a cutoff of >16 mmHg for the obese group, considering the necessary adjustments for possible confounders.

With respect to ND, in the group of obese patients, 73% of patients with elevated MS showed a physiological ND (drop in BP greater than 10% for this period). In contrast, in the group of non-obese patients, ND associated with the increase in MS was seen in 66.7% (Table 3). Also, and as expected, the number of anti-hypertensive agents used in the group of obese patients was higher than in the non-obese group (Table 4).

**Table 3:** – Association between the presence of morning surge (MS) and nocturnal dipping pattern in the studied groups

	Nocturnal dipping pattern	Absence of morning surge	Presence of morning surge	p-value

		n (%)	n (%)	
Não obesos	Present	24 (43.6)	36 (66.7)	**0.03**
	Attenuated	22 (40.0)	15 (27.8)	
	Reverse	9 (16.4)	3 (5.5)	
Obesos	Present	25 (43.9)	27 (73.0)	**0.02**
	Attenuated	24 (42.1)	8 (21.6)	
	Reverse	4 (7.0)	2 (5.4)	

Nocturnal dipping was stratified into a) present (nocturnal blood pressure dipping between 10% and 20% relative to daytime); b) attenuated (nocturnal blood pressure dipping of 0-10%); c) reverse (nighttime blood pressure greater than daytime blood pressure).

**Table 4:** – Number of anti-hypertensive drugs between the studied groups

Groups and drugs							

		Number of medications					Total

		0	1	2	3	4	
Group	Non-obese	31	21	36	13	8	109
	Obese	12	7	30	31	14	94
**Total**		43	28	66	44	22	203

Number of anti-hypertensive drugs by group (obese and non-obese).

## Discussion

Our study was designed to determine the prevalence of MS in obese hypertensive patients who had echocardiographic evidence of LVH, in accordance with guidelines on the management of hypertension and prevention of complications.^18,19^ Morning BP surge is an easy-to-measure BP-related parameter, consisting of the difference of mean BP between awakening and sleep period.

The prevalence of obesity in the industrialized world has increased to alarming levels in the last decades. It has been estimated that a significant proportion of hypertension cases are associated with obesity, and that obese individuals are more likely to develop hypertension. Obesity, hypertension and LVH are important cardiovascular risk factors. Thus, new biomarkers have been investigated for the prediction of major cardiovascular outcomes and even surrogate outcomes.

Normally, there is a decrease in BP during sleep in relation to wakefulness. The sudden increase in BP in the transition from sleep to awake is known as MS. Vascular mechanisms that lead to excessive morning BP surge and its pathophysiological implications have not been fully elucidated, with the first evidence of the relationship between MS and cardiovascular events being reported in 2003 by Kario et al.,^20^ who observed an independent association with silent cerebrovascular disease. In the Ohasama study, conducted by Metoki et al.,^21^ a positive significant association between MS and hemorrhagic stroke was reported.^21^ In addition, the relationship between MS and cardiovascular outcomes was reported in another trial, which investigated this association in patients with ND.^22^ Li et al.,^23^ evaluating an extensive database (International Database on Ambulatory Blood Pressure) with 5,645 participants from eight countries, showed that MS is predictive of cardiovascular events, particularly stroke in Asians and coronary events in Europeans.^23^ The study by Pierdomenico et al.^24^ showed that high MS predicts stroke in elderly patient treated for hypertension, who have ND.^24^ In contrast, in the study by Verdecchia et al.^25^ with 3012 initially untreated subjects, those allocated in the upper quartile of MS showed the lowest risk for major cardiovascular events.^25^ Discrepancy in these results is probably due to numerous factors including the heterogeneity of populations, confounders, and lack of a specific cut-off point for high MS. Mean age, prevalence of hypertension and anti-hypertensive therapy, follow-up duration, evaluation of the impact of ND and ethnics tended to differ between the studies. A systematic review and meta-analysis pooled data on MS from 14133 individuals of seven longitudinal studies, with a mean follow-up of 7,1 years. It was shown that excess MS was associated with increased risk of overall mortality. Patients with high MS showed a tendency for increased risk of overall mortality, stroke and cardiovascular events, but with no statistical difference.^26^ A meta-analysis conducted by Sheppard et al.^27^showed that, when analyzed as a continuous variable, which has more power to detect an association, a 10mmHg increase in MS was associated with increased risk of stroke.^27^ Similar results were reported by the previously mentioned study by Kario et al.,^20^ who evaluated MS as a continuous variable, and showed that increases in this biomarker was associated with increased risk of stroke.

A universal cut-off value for abnormal MS has not been established yet. stroke. Hoshide et al.,^28^ in the ARTEMIS study, showed that MS was higher in Japanese than in European hypertensive patients, even after adjusting for age and 24-hour mean BP levels. This difference remained significant after accounting for differences in ND.^28^ Markers of hypertensive cardiac disease, which includes increases in LVMI, LVH and a lower A/E ratio (measurement of diastolic dysfunction) were associated with excess morning BP surge.^29-32^

Difficulties in body weight reduction and drug therapy management to achieve treatment adherence and minimize degenerative chronic diseases are the main challenges of the multidisciplinary team. To identify instruments or indicators of associations may be useful in predicting events, notably in the obese hypertensive population. Obesity should no longer be considered merely as a risk factor, but rather a chronic disease and a public health problem, requiring investments in researches and treatment in the following years. In addition, excess body fat, currently considered an independent risk factor for cardiovascular diseases, is a predisposing factor for the development of hypertension in these patients. Due to the increasing number of hypertensive individuals in the world, epidemiological studies on BP have been increasingly discussed, considering significative correlations between BP variability (indicators like the type of MS and NS, among others) and the development of target-organ damage.

Most physiological mechanisms follow a circadian pattern, determined by a complex interaction of our “biological clock” with environmental and behavioral factors. Many of these mechanisms have a direct effect on the cardiovascular system and contribute to the increase in BP. Changes in autonomous nervous system activity, notably those related to an increased sympathetic activity, seem to be the main underlying factor in MS.^33^ Wanthong et al.^34^ described that BP levels on awakening were correlated with LVMI and with residual risk for cardiovascular events. Besides, the authors showed the importance of the sleep BP as an independent maker of organ damage.^34^

In our study population, we found that in the obese hypertensive group, MS greater than 16mmHg showed a positive linear correlation with LVH, and this cut-off point was the one that defined the best performance of sensitivity and specificity. Probably, and speculatively, obesity either potentializes the aggressive effects of MS or it acts as an adjuvant to the risk. The coexistence of obesity and hypertension increases the probability of cardiovascular complications, whereby excess body weight increases the risk for other diseases like diabetes and chronic kidney disease.^35^ In fact, in our study group, we found that 46.8% of the obese population were also diabetic in contrast with 17.4% in the non-obese group (p<0.01). Nearly 33% of non-obese patients (group 1) with LVH and diabetes had MS, versus 80% in the obese group.

There is evidence that BP measures determined by ABPM are better predictors of the outcomes, including overall mortality, for their accuracy as compared with office BP measures.^36^ In our study, mean BP was 16.1±12.6 mmHg in obese hypertensive individuals and 22.1±13.3 mmHg (p<0.001) in non-obese hypertensive individuals. We highlight the positive association between MS > 16 mmHg and the presence of LVH in the obese group, and speculate that a lower MS may be used and stratified for this population.

Despite little available evidence on MS in obesity, Amodeo et al.^37^indicated the need for more population studies to evaluate the impact of MS, and to define a cut-off point for MS.^37^In our cohort, MS values above 16mmHg had the strongest association with LVH in the group of obese individuals. Therefore, we found that obesity may have a marked influence on BP values and left ventricular structural changes. In obese hypertensive individuals, mean LVMI was higher as compared with non-obese hypertensive individuals. In a prospective study^38^ with 433 participants, obesity and hypertension was the main determinants of left ventricular remodeling, and development of LVH. By a significant interaction between obesity and hypertension, obese individuals usually develop hypertension and pressure overload, with an exponential effect on the prevalence of LVH. Large epidemiological studies showed that hypertension was crucial factor for left ventricular concentric remodeling and concentric left ventricular hypertrophy, independently of obesity.^38-40^ Besides, there is evidence that macrophage migration inhibitory factor, a cytokine involved in a wide range of events in the immune system, would be associated with endothelial dysfunction and left ventricular remodelling.^41^

Therefore, in hypertensive obese subjects, we believe that MS may be an associated factor and show a particular behavior, and morning BP measurements could be more sensitive to detect the impact of BP variability on cardiovascular risk. Based on the analysis of our observations, we recommend the development of an optimized strategy that includes the measurement of BP parameters and speculate that MS, especially in specific populations, like obese hypertensive patients, may be associated with myocardial structural changes.

## Conclusion

In obese hypertensive patients, high MS was positively associated with LVH, with a correlation threshold lower than that observed in the non-obese patients. This analysis of BP by ABPM revealed that morning BP values measured at early morning were associated with target-organ damage, particularly LVH. Therefore, our findings may be useful in assessing the residual risk in subgroups of patients despite and in addition to the risk assessed by conventional BP measurement.

### Study limitation

Similar to population studies, the difficulty in detecting morning BP surge in obese individuals lies in obtaining ideal measurements, due to inappropriate cuff size, and possible complications in this period. As this was an observational study, causality between the studied variables could not be inferred. Both sleeping and waking times were self-reported and the possibility that these times were erroneously reported by some patients cannot be excluded, resulting in possible errors in estimating morning BP surge. Another possible limitation is that we did not analyze other variables, such as sleep quality and maybe a larger sample, and we did not assess external validity of our findings.
